# Septic shock due to *Yersinia pseudotuberculosis* infection in an adult immunocompetent patient: a case report and literature review

**DOI:** 10.1186/s12879-020-05733-w

**Published:** 2021-01-07

**Authors:** Takehiro Hashimoto, Ryuichi Takenaka, Haruka Fukuda, Kazuhiko Hashinaga, Shin-ichi Nureki, Hideki Hayashidani, Teruo Sakamoto, Osamu Shigemitsu

**Affiliations:** 1 Department of Respiratory Medicine and Infectious Diseases, Oita University Faculty of Medicine, 1-1 Idaigaoka, Hasama-machi, Yufu, Oita 879-5593 Japan; 2 Department of Emergency Medicine, Oita University Faculty of Medicine, 1-1 Idaigaoka, Hasama-machi, Yufu, Oita 879-5593 Japan; 3 Advanced Trauma, Emergency, and Critical Care Center, Oita University Hospital, 1-1 Idaigaoka, Hasama-machi, Yufu, Oita 879-5593 Japan; 4 Division of Animal Life Science, Institute of Agriculture, Tokyo University of Agriculture and Technology, 3-5-8 Saiwai-cho, Fuchu, Tokyo, 183-8509 Japan

**Keywords:** Azithromycin, Bacteremia, Cefmetazole, Ceftriaxone, Doripenem, Septic shock, *Yersinia pseudotuberculosis*

## Abstract

**Background:**

*Yersinia pseudotuberculosis* infection can occur in an immunocompromised host. Although rare, bacteremia due to *Y. pseudotuberculosis* may also occur in immunocompetent hosts. The prognosis and therapeutic strategy, especially for immunocompetent patients with *Y. pseudotuberculosis* bacteremia, however, remains unknown.

**Case presentation:**

A 38-year-old Japanese man with a mood disorder presented to our hospital with fever and diarrhea. Chest computed tomography revealed consolidation in the right upper lobe with air bronchograms. He was diagnosed with pneumonia, and treatment with intravenous ceftriaxone and azithromycin was initiated. The ceftriaxone was replaced with doripenem and the azithromycin was discontinued following the detection of Gram-negative rod bacteria in 2 sets of blood culture tests. The isolated Gram-negative rod bacteria were confirmed to be *Y. pseudotuberculosis*. Thereafter, he developed septic shock. Doripenem was switched to cefmetazole, which was continued for 14 days. He recovered without relapse.

**Conclusions:**

We herein report a case of septic shock due to *Y. pseudotuberculosis* infection in an adult immunocompetent patient. The appropriate microorganism tests and antibiotic therapy are necessary to treat patients with *Y. pseudotuberculosis* bacteremia.

## Background

*Yersinia pseudotuberculosis* is a Gram-negative rod bacterium belonging to the family Enterobacteriaceae. *Y. pseudotuberculosis* is commonly found in contaminated food and water [1]. Transmission to humans is uncommon and occurs through the ingestion of contaminated food, water or milk, or direct contact with an infected animal, such as rodents, rabbits, deer, farm animals, and birds [1, 2]. *Y. pseudotuberculosis* infections typically manifest gastroenteritis [2]. A self-limiting acute infection is common. Severe infections and chronic conditions can also occur, however, particularly in immunocompromised patients. Although rare, *Y. pseudotuberculosis* infection may progress to bacteremia in adult immunocompetent patients. The definition of septic shock was changed to be more strict in 2016 [3]. In the literature, there are no case reports fulfilling the new definition of septic shock in adult immunocompetent patients with *Y. pseudotuberculosis* infection. We herein report the first case of septic shock due to *Y. pseudotuberculosis* infection in an adult immunocompetent patient. The patient was successfully treated with the appropriate antibiotics.

## Case presentation

**Fig. 1:**
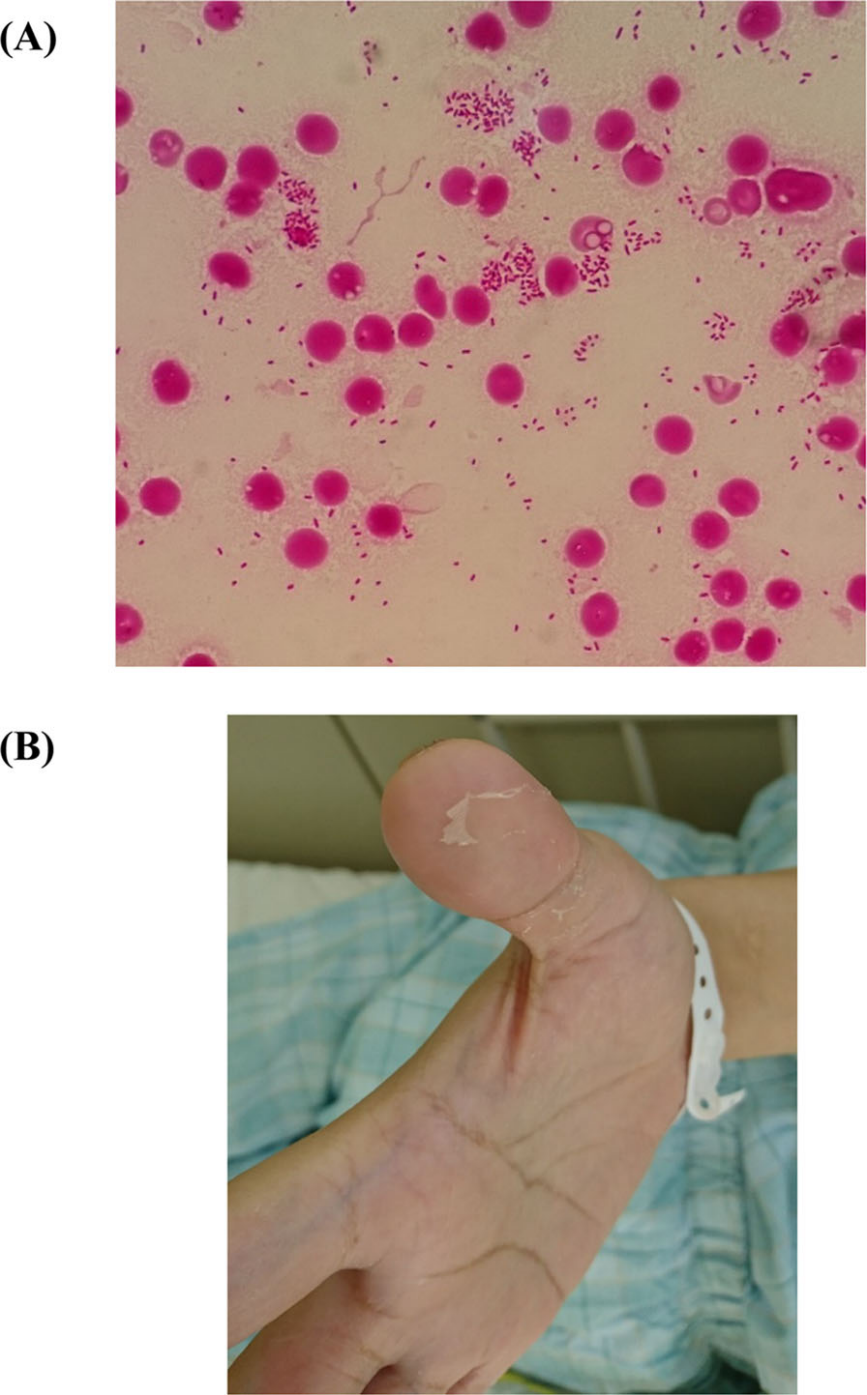
**a** Blood culture test detected Gram -negative rod bacteria (Gram stain, magnification 1000 ×) on day 3. **b** Desquamation of the fingers was observed on day 7

A 38-year-old Japanese man with a mood disorder was admitted to the emergency department of Oita University Hospital (Oita, Japan) for complaints of fever and diarrhea. Ten days prior to presentation at the emergency department, he experienced appetite loss and vomiting a few days after attending a riverside barbecue and eating half-roasted foods. He was receiving oral ethyl loflazepate and paroxetine hydrochloride hydrate therapy for mood disorder and alcoholism. The patient had a fever and watery diarrhea 3 days prior to admission. His body temperature was 38.8 °C, blood pressure 111/70 mmHg, pulse 112 beats/min, and he had an SpO_2_ of 100% at 3 L/min with a nasal mask on admission. Although he was awake without any stimuli, he was unable to recall his name or date of birth. No murmur was detected on heart examination. No crackles were auscultated in either lung field. Laboratory tests revealed an elevated white blood cell count (11,840/μL), hypoalbuminemia (2.6 g/dL), decreased serum iron level (22 μ/dL), decreased serum unsaturated iron-binding capacity level (162 μg/dL), decreased transferrin saturation (TSAT) level (12%), elevated C-reactive protein level (9.58 mg/dL), and an elevated procalcitonin (18.8 ng/mL). A chest X-ray showed an infiltrative shadow in the right upper lung field. Whole body computed tomography showed no remarkable finding except for consolidation with air bronchograms in the right upper lung lobe. The patient was diagnosed with community-acquired pneumonia, and treatment with intravenous ceftriaxone (2 g every 24 h) and azithromycin (500 mg every 24 h) was initiated. On day 3, 2 sets of blood culture tests revealed the presence of Gram -negative rod bacteria (Fig. 1-a); therefore, the ceftriaxone was replaced with doripenem (500 mg every 8 h) and the azithromycin was discontinued. On day 4, the Gram-negative rod bacteria were identified as *Y. pseudotuberculosis*, using the VITEK 2 system (bioMérieux, Marcy l’Etoile, France) with 99% probability. Furthermore, matrix-assisted laser-desorption/ionization time-of-flight mass spectrometry (Bruker Daltonics, Billerica, MA, USA) was used to identify the bacteria as *Y. pseudotuberculosis* with highly probable species identification (score value, 2.32). On day 5, the patient’s blood pressure decreased to 80/50 mmHg (mean arterial pressure 60 mmHg). Blood gas analysis revealed an elevated lactate level (4.1 mmol/L). The hypotension persisted despite adequate volume resuscitation, and norepinephrine as a vasopressor was started. The patient was diagnosed with septic shock due to *Y. pseudotuberculosis* infection. On the basis of susceptibility testing of *Y. pseudotuberculosis* using a dry plate (Eiken, Tokyo, Japan) with a conventional microdilution method and analysis by an image analyzer (Koden IA40MIC-i, Koden, Tokyo, Japan), the strain was found to be sensitive to ampicillin, ampicillin/sulbactam, cefotiam, ceftazidime, cefmetazole, meropenem, gentamicin, and levofloxacin. A sputum culture test was negative. A stool culture test detected *Escherichia coli* and *Streptococcus spp,* but no *Y. pseudotuberculosis.* On day 7, his fever was reduced and the norepinephrine was discontinued. Desquamation of the fingers was observed (Fig. 1-b). Two sets of blood culture tests showed negative results. On day 9, the treatment regimen was changed to cefmetazole (2 g every 8 h) and continued for a total of 14 days. On day 10, a chest X-ray showed complete improvement of the infiltrative shadow in the right upper lung field. On day 17, he was discharged from the hospital. Further characteristics of the *Y. pseudotuberculosis* strain was performed because of the clinical importance of virulence factors of this strain. *Y. pseudotuberculosis* isolate was of serotype 6 using serotyping scheme based on O-antigen. To evaluate the pathogenicity of this isolate, variants of *Y. pseudotuberculosis*-derived mitogen (YPM) superantigens were investigated in the strain using PCR with the following primers: ypmA [(5′-CACTTTTCTCTGGAGTAGCG-3′ (forward) and 5′-GATGTTTCAGAGCTATTGTT-3′ (reverse)] and ypmB [(5′-TTTCTGTCATTACTGACATTA-3′ (forward) and 5′-CCTCTTTCCATCCATCTCTTA-3′ (reverse)] [4]. The PCR test using isolated bacteria showed positivity of ypm A. Combined with the results of serotype 6 and genetic detection of *Yersinia pseudotuberculosis*-derived mitogen A (YPMa), *Y. pseudotuberculosis* strain belonged to a genetic group 3 (Far East systemic pathogenicity type) [5]. We finally diagnosed with Far East scarlet-like fever (FESLF) caused by *Y. pseudotuberculosis* complicated septic shock.

## Discussion and conclusions

We report a case of septic shock due to *Y. pseudotuberculosis* infection in an adult immunocompetent patient. *Y. pseudotuberculosis* infection in humans was first described in 1883 [6]. It is a Gram-negative rod bacterium and can grow in temperatures as low as 4 °C [2]. The incubation period for intestinal *Y. pseudotuberculosis* infection is approximately 3 to 7 days [7]. Cases of *Y. pseudotuberculosis* infection, including gastroenteritis, pseudoappendicitis, bacteremia, pharyngitis, erythema nodosum, reactive arthritis, and syndromes mimicking Kawasaki disease, have been reported [2, 5, 8]. Risk factors for *Y. pseudotuberculosis* infection are exposure to contaminated food or water, as well as underlying medical conditions such as hepatic cirrhosis, HIV infection, malignancy, anaplastic anemia, thalassemia, iron overload, and diabetes mellitus [9, 10]. The mortality of bacteremia due to *Y. pseudotuberculosis* is reported to be as high as 75% [11]. Nine cases of *Y. pseudotuberculosis* bacteremia in adult immunocompetent patients were reported from 1911 through 1994 [2]. Of the 9 patients, 6 (66%) died. Four cases reported from 1995 to 2020 and our case of *Y. pseudotuberculosis* bacteremia in an adult immunocompetent patient are summarized in Table 1 [12–15]. All 5 patients survived. Although the case numbers of *Y. pseudotuberculosis* bacteremia in adult immunocompetent patients are limited, the prognosis of recent cases of *Y. pseudotuberculosis* bacteremia in adult immunocompetent patients might be better than that of the previous cases. The better outcome may be related to advances in diagnostic techniques and antibiotic therapy. Further studies are required to establish the prognosis of *Y. pseudotuberculosis* bacteremia in adult immunocompetent patients. Alcohol consumption is known to result in an iron overload, which could be a predisposing factor for systemic *Y. pseudotuberculosis* infection. Although serum ferritin level was not examined in our case, low serum iron and TSAT levels implied the low possibility of iron overload. Thus, we considered the patient would be immunocompetent.

**Table 1:** Cases of *Yersinia pseudotuberculosis* bacteremia in adult immunocompetent patients. “Septic shock” is defined based on a clinical construct of sepsis with persistent hypotension requiring vasopressors to maintain a mean arterial pressure of ≥65 mmHg and having a serum lactate level > 2 mmol/L despite adequate volume resuscitation

Author	Age/Sex	Probable portal of entry	Contaminated food or water exposure	Septic shock	Treatment	Outcome	Reference
Ljungberg et al.	54/M	Unknown	Unknown	No	PCG→ CTRX→ CPFX	Survived	[12]
Ressler et al.	68/F	Skin and soft tissue	Unknown	No	Unknown	Survived	[13]
Lai et al.	33/M	Gastrointestinal tract	Unknown	Unknown	MFLX→ CPFX	Survived	[14]
Mashiba et al.	22/F	Unknown	None	No	CEZ→ FOM→ IPM/CS	Survived	[15]
Our case	38/M	Gastrointestinal tract	Undercooked food	No	CTRX→ DRPM→ CMZ	Survived	

Age (years old) and sex (F, female; M, male). *Abbreviations*: *CEZ* cefazolin, *CMZ* cefmetazole, *CPFX* ciprofloxacin, *CTRX* ceftriaxion, *DRPM* doripenem, *FOM* fosfomycin, *IPM/CS* imipenem/cilastatin, *MFLX* moxifloxacin, *PCG* penicillin G

FESLF caused by *Y. pseudotuberculosis* infection is a severe inflammatory disease that occurs sporadically and outbreaks in Russia and Japan [5]. FESLF patients with *Y. pseudotuberculosis* infection can be complicated with desquamation at the distal portion of the extremities in convalescent phase [16, 17]. Desquamation is seen in 83% of *Y. pseudotuberculosis* infection cases in childhood [17]; however, few cases have reported desquamation as a complication of *Y. pseudotuberculosis* infection in adults [16, 18]. Further studies are required to establish the epidemiology of desquamation in *Y. pseudotuberculosis* infection in adults.

In our case, *Y. pseudotuberculosis* strain was of serotype 6 and harbored superantigen gene ypmA. *Y. pseudotuberculosis* has been classified into serotypes 1 to 15 [5]. Most European *Y pseudotuberculosis* isolates are of serotypes 1 to 3, whereas serotypes 4 to 15 are primarily found in Asia [5]. YPMa is a superantigenic toxin produced almost by Far Eastern strains [4] which is involved in the pathogenesis of severe inflammatory disease from patient with FESLF. Thus, the clinical isolate from our patient was compatible with the that from FESLF patients.

The definitions of sepsis and septic shock were changed in 2016 [3]. Septic shock can be defined with persistent hypotension requiring vasopressors to maintain a mean arterial pressure of ≥65 mmHg and a serum lactate level > 2 mmol/L despite adequate volume resuscitation. In the previous diagnostic criteria, septic shock was defined as sepsis with hypotension despite adequate fluid resuscitation [19]. Thus, septic shock according to the new definition is more critical than that based on the previous criteria. Lai et al. reported a case of *Y. pseudotuberculosis* infection in an adult patient who developed to septic shock [14], but their case was reported before 2016 and it is unclear whether their case fulfilled the new septic shock criteria [3]. To our knowledge, according to the new septic shock criteria, our case could be the first case of septic shock due to *Y. pseudotuberculosis* infection in an adult immunocompetent patient.

*Y. pseudotuberculosis* exhibits greater susceptibility to antimicrobials other than macrolides. In a murine model, fluoroquinolone therapy is effective against *Y. pseudotuberculosis* infections whereas beta lactam therapy is associated with lower survival or a poor clinical response [20, 21]. The best antimicrobial therapy for *Y. pseudotuberculosis* infection, however, is not yet established. Further studies are needed to determine the appropriate treatment for *Y. pseudotuberculosis* infections with bacteremia.

In our case, the illness developed a few days after the patient attended a barbecue. For prevention, it is important to take into account whether or not contaminated food and water were consumed raw and to exclude the possibility of secondary contamination due to undercooked food and unboiled water. Although stool culture did not detect *Y. pseudotuberculosis* in our case, bloodstream infection could originate from intestinal infection. Vulnerability of intestinal tract leading to bacteremia might be caused by mucosal damage due to inflammation of *Y. pseudotuberculosis* infection and intestinal edema came from hypoalbuminemia.

In conclusion, we herein report a case of septic shock due to *Y. pseudotuberculosis* infection in an adult immunocompetent patient. This report will help to raise awareness among clinicians that *Y. pseudotuberculosis* bacteremia should be included in the differential diagnosis when patients exhibit fever and diarrhea after consuming undercooked food.

## Data Availability

All the data on clinical findings are included in the manuscript.

## Abbreviation

*Y. pseudotuberculosis*: Y*ersinia pseudotuberculosis*
